# Advantages of an Improved Rhesus Macaque Genome for Evolutionary Analyses

**DOI:** 10.1371/journal.pone.0167376

**Published:** 2016-12-02

**Authors:** Julien S. Gradnigo, Abhishek Majumdar, Robert B. Norgren, Etsuko N. Moriyama

**Affiliations:** 1 School of Biological Sciences, University of Nebraska-Lincoln, Lincoln, Nebraska, United States of America; 2 Department of Genetics, Cell Biology and Anatomy, University of Nebraska Medical Center, Omaha, Nebraska, United States of America; 3 School of Biological Sciences and Center for Plant Science Innovation, University of Nebraska-Lincoln, Lincoln, Nebraska, United States of America; Midwestern University, UNITED STATES

## Abstract

The rhesus macaque (*Macaca mulatta*) is widely used in molecular evolutionary analyses, particularly to identify genes under adaptive or unique evolution in the human lineage. For such studies, it is necessary to align nucleotide sequences of homologous protein-coding genes among multiple species. The validity of these analyses is dependent on high quality genomic data. However, for most mammalian species (other than humans and mice), only draft genomes are available. There has been concern that some results obtained from evolutionary analyses using draft genomes may not be correct. The rhesus macaque provides a unique opportunity to determine whether an improved genome (MacaM) yields better results than a draft genome (rheMac2) for evolutionary studies. We compared protein-coding genes annotated in the rheMac2 and MacaM genomes with their human orthologs. We found many genes annotated in rheMac2 had apparently spurious sequences not present in genes derived from MacaM. The rheMac2 annotations also appeared to inflate a frequently used evolutionary index, ω (the ratio of nonsynonymous to synonymous substitution rates). Genes with these spurious sequences must be filtered out from evolutionary analyses to obtain correct results. With the MacaM genome, improved sequence information means many more genes can be examined for indications of selection. These results indicate how upgrading genomes from draft status to a higher level of quality can improve interpretation of evolutionary patterns.

## Introduction

Genome projects consist of assemblies of sequences and annotation of gene feature information within these sequences. Errors can occur in the initial sequencing or assembly of sequences [1, 2]. When automated annotators are applied to imperfectly assembled sequence data, additional errors may occur [3]. For example, when an exon sequence is missing from an assembly, automated annotation pipelines such as the one used by the National Center for Biotechnology Information (NCBI) [4] will frequently select an intronic sequence to annotate as the missing exon [3]. This error results in incorrect protein models [1, 2]. Furthermore, previous studies have indicated that errors in draft genomes distort the results of molecular evolutionary analyses [1, 5–8].

To understand human evolution, it is necessary to compare human genes with those of other non-human primates. A draft assembly for rhesus macaques, *Macaca mulatta*, was produced in 2007 [9]. This assembly, rheMac2, has been annotated by the NCBI Eukaryotic Genome Annotation Pipeline, which includes the Gnomon gene prediction method. We and other researchers have identified errors in sequence, assembly, and annotation in this genome [2, 3, 10, 11]. We recently produced a new rhesus macaque assembly and annotation, MacaM [12]. On a variety of metrics, including completeness of the assembly and accuracy of annotation, MacaM was a substantial improvement with respect to rheMac2 assembly and annotation [12]. Specifically, 50% of the NCBI annotations for rheMac2 were missing, incomplete, or wrong [2]. This was in part due to misassemblies of rheMac2 [2] and in part due to errors made by automated annotation pipelines [3, 12]. We demonstrated that MacaM was more complete than rheMac2 by standard measures such as contig and scaffold N50 and empirical methods such as aligning Ion Torrent genomic reads against the two assemblies [12]. Most importantly, we demonstrated that the MacaM protein models were far more likely to be correct than those derived from rheMac2 [12]. The rheMac2 assembly was annotated independently by Ensembl. We demonstrated that this annotation also showed errors similar to those produced by Gnomon (see Fig 3 in [12]).

The improved assembly and annotations available for MacaM should facilitate more accurate and complete molecular evolutionary studies. To test this hypothesis, we examined the apparent evolution of proteins by comparing the coding sequences derived from rheMac2 and MacaM annotations with corresponding human orthologous sequences. Specifically, we cataloged errors in gene models that resulted in apparently spurious sequences in rheMac2 annotations that could be misinterpreted as species-specific. Such errors were relatively rare in MacaM. Furthermore, use of the improved rhesus genome (MacaM) largely avoided the inflation of ω, the ratio of nonsynonymous to synonymous substitution rates, observed when rheMac2 was used. Our analyses demonstrated how an improved genome can lessen errors that could affect the interpretation of evolutionary patterns. Most importantly, with the high quality MacaM genome, evolutionary analysis can be performed for many genes that would have had to be filtered out using laborious ad hoc processes previously. Our findings argue for increased allocation of resources to improve existing draft genomes to higher levels of quality rather continuing to produce yet more draft genomes.

## Material and Methods

### Selection of human-rhesus ortholog sets

Protein-coding genes were identified, and coding sequences extracted from the rheMac2 assembly using NCBI’s annotation (GFF file) [13]. We chose to use the GFF file NCBI provides as annotation for rheMac2 as this is relatively stable and corresponds to the rheMac2 assembly. Note that sequences currently found in NCBI/GenBank for rhesus macaque genes may differ from the GFF annotations, since nucleotide and protein sequences in GenBank are constantly being revised based on multiple types of information such as expressed sequence tags, cDNA sequencing, transcriptome assemblies, and models derived from genome annotations. To determine the potential advantages of improving a draft mammalian genome for evolutionary analyses, we used the MacaM genome (version 7 assembly, version 7.6.8 annotation), which is described in [12] (its most recent release is available at [14]). To extract the coding sequences from each rhesus genome, the gffread utility of Cufflinks (version 2.0.2) [15] as well as our own in-house program were used. Human genes for which only a single transcript has been reported were previously identified as part of the MacaM genome annotation [12]. For this study, we chose 3,606 genes for comparison that met two criteria: 1. They were annotated in both rheMac2 and MacaM (to facilitate direct comparisons) and 2. Human orthologs were reported to have a single isoform (to reduce the possibility of pairing non-orthologous transcript forms incorrectly). Of these 3,606 genes, 1,131 had different coding sequences in rheMac2 and MacaM. The 3,606 orthologs are listed in S1 Table.

For further analyses, we downloaded the coding sequences of the 3,606 genes from the Mmul_8.0.1 assembly (GCF_000772875.2, Annotation ID 102) from NCBI.

### Molecular evolutionary analysis

For each gene set, the two rhesus sequences were individually aligned against the human ortholog sequence. We first aligned proteins using MAFFT (v.7.050b) with the L-INS-i option [16]. Alignments of coding nucleotide sequences corresponding to their protein alignments were generated using TranslatorX [17]. Each coding sequence alignment was preprocessed for further evolutionary analysis using PAL2NAL [18] by removing stop codons and gaps from each pairwise alignment. The numbers of synonymous and nonsynonymous substitutions per site (dS and dN, respectively) and ω (dN/dS) were estimated by the method of Yang and Nielsen [19] implemented in the program yn00, which is part of the package PAML (Phylogenetic Analysis by Maximum Likelihood, version 4.7) [20].

All statistical analyses were performed with JMP Pro 12.1.0 statistics software (SAS Institute Inc.).

## Results

**Fig 1:**
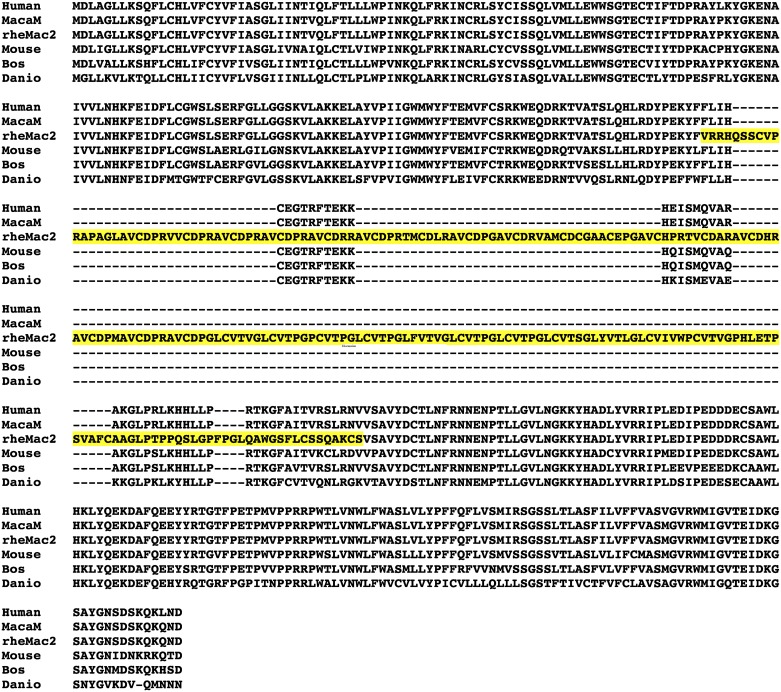
Alignment of the AGPAT4 protein sequences among five vertebrates. Two versions of the rhesus AGPAT4 protein are aligned against the AGPAT4 orthogs from human and three vertebrates. The potentially incorrect sequence regions are shown in yellow background. Accession numbers: NP_064518.1 (*Homo sapiens*, human), NP_080920.2 (*Mus musculus*, mouse), NP_001015537 (*Bos taurus*, cattle), and NP_998157.1 (*Danio rerio*, zebrafish).

**Fig 2:**
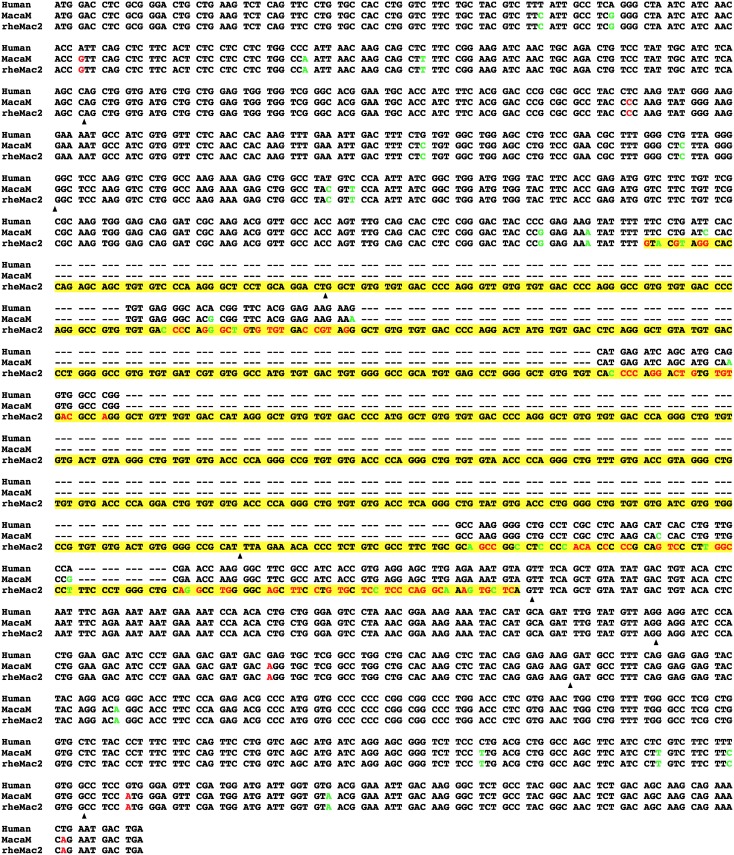
An example of missannotation and missalignment with the rheMac2/NCBI annotation. Two versions of the rhesus AGPAT4 coding sequence are aligned against the AGPAT4 orthog from human (NM_020133.2). The potentially incorrect sequence regions are shown in yellow background. Exon-intron boundaries given in the rheMac2/NCBI annotation are demarcated with black arrowheads. Nucleotide positions where rhesus sequences have synonymous and nonsynonymous substitutions compared to the human sequence are indicated with green and red fonts, respectively.

We observed striking differences between some rheMac2/NCBI and MacaM alignments with human ortholog coding sequences. These differences were of two general classes: 1. extensive areas of nonalignment (gaps) between rheMac2/NCBI and human ortholog sequences and 2. significant differences in both nucleotide and protein sequences in some aligned regions of rheMac2/NCBI and human orthologs. A detailed analysis of one rheMac2 /NCBI gene model illustrating these two general classes is displayed in Figs 1 and 2. In humans, AGPAT4 (1-acylglycerol-3-phosphate O-acyltransferase 4, or 1-acylglycerol-3-phosphate O-acyltransferase delta) codes for a 378 amino-acid (aa) long protein (mRNA: NM_020133.2, protein: NP_064518.1). Its protein sequence is highly conserved among vertebrates (*e*.*g*., 80% similarity with the zebrafish protein; Fig 1). However, the rheMac2/NCBI AGPAT4 ortholog includes ~200 aa of a nonalignable region. Fig 2 shows the alignment of the human AGPAT4 coding sequence with the two versions of the rhesus AGPAT4 coding sequence. The MacaM gene model of the AGPAT4 gene has a protein sequence that is 99% identical to the human AGPAT4 protein without any insertions or deletions. In contrast, the rheMac2/NCBI version includes three incorrectly identified exons in the middle of the gene (colored in yellow in Fig 2). When a protein similarity search was conducted using the human AGPAT4 protein sequence as the query against the *M*. *mulatta* RefSeq protein database, due to this erroneously inserted region, one of its paralogs, AGPAT3 (XP_001104367), was returned as the top hit (77% similarity, E = 1x10^-146^). The correct AGPAT4 ortholog was returned as the second hit (E = 6x10^-117^). The AGPAT4 gene encompasses a 138,100 bp region in the scaffold NW_001116526.1. In the region between the fourth and fifth exons the scaffold sequence includes 200 bp of an assembly gap (filled with 'n's), which likely contributed to the incorrect gene model provided by NCBI for the rheMac2 assembly. The NCBI database includes the protein entry EHH18819, which is derived from a Chinese rhesus macaque genome produced by the Beijing Genomics Institute (BGI) [21]. This protein sequence is 100% identical to the MacaM sequence for AGPAT4 (although it is annotated as a “hypothetical protein”). It is worth noting that the chimpanzee AGPAT4 ortholog in NCBI RefSeq (XM_009452365 and XP_009450640.1) has the same error found in the rheMac2/NCBI annotation.

The coding sequence alignment in Fig 2 demonstrates that many nucleotides in the rheMac2/NCBI version differ from both the human and MacaM nucleotides, especially nonsynonymous changes (indicated with red letters). These differences greatly increase the dN as well as the ω estimates for the rheMac2/NCBI version (dS = 0.1996, dN = 0.0865, and ω = 0.4336) compared to those for the MacaM version (dS = 0.0794, dN = 0.0071, and ω = 0.0894).

To quantify the effect of differences in annotation between rheMac2/NCBI and MacaM for a large-scale, genome-wide analysis, we compared the 3,606 gene set using each of these rhesus annotations with human orthologs. Many rhesus genes had significantly different lengths compared to human orthologs when the rheMac2 genome was used. The mean length difference was only -0.21±11.40bp (ranging from -567 to 147bp) with the MacaM genome, while with the rheMac2 genome, the mean difference was -1.76±383.45bp (ranging from -8,103 to 5,448bp) (S1 Table).

**Fig 3:**
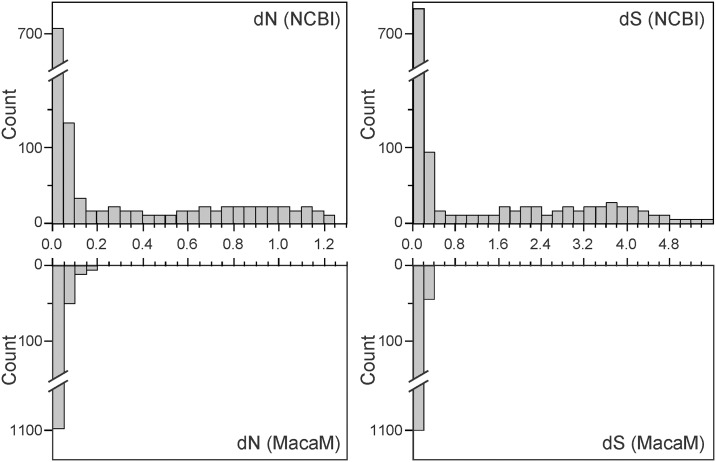
Distributions of nonsynonymous (dN) and synonymous (dS) rates estimated between human and rhesus macaque orthologs. Frequency distributions of dN (left panels) and dS (right panels) are shown for the 1,131 genes where the two rhesus genome annotations have different coding sequences. The top panels show the distributions obtained using the rheMac2/NCBI annotation and the bottom panels show those obtained using the MacaM annotation.

**Fig 4:**
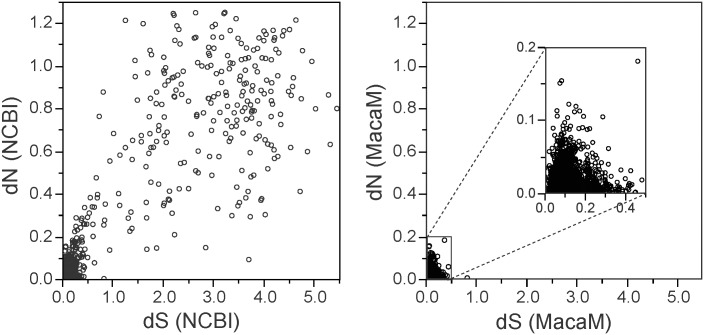
Comparison of dN and dS estimated from human and rhesus macaque orthologs when two rhesus genome annotations are used. dN and dS values are plotted for a comparison of human and the two rhesus annotations, rheMac2/NCBI (left panel) and MacaM (right panel), for 3,606 orthologs. An enlarged view of the boxed area at the lower left corner of the right panel is shown in the inset.

To determine the effect of improving the rhesus genome on genome-wide evolutionary analysis, we calculated dN, dS, and ω from each ortholog pair. Significantly higher substitution rates (both dS and dN) were observed with the rheMac2/NCBI sequences than with the MacaM sequences (Fig 3). For 1,131 genes whose rhesus coding sequences were different between the two annotations, the mean dNs were 0.196±0.332 for the rheMac2/NCBI sequences and 0.014±0.016 for the MacaM sequences. The mean dSs were 0.728±1.275 for the rheMac2 /NCBI sequences and 0.082±0.049 for the MacaM sequences. These distributions were found to be significantly different (*P* < 0.0001 by both two-tailed *t*-test and Mann-Whitney *U* test). A more granular analysis is shown in Fig 4 where dN and dS values are plotted for individual genes. When the rheMac2/NCBI sequences were used (left panel), a significant number of genes appeared to have high sequence divergence. Such apparent divergences were not seen when the MacaM annotation was used (Fig 4, right panel). Both dN and dS were significantly larger when rheMac2/NCBI sequences were used as the source for rhesus macaque sequences than when MacaM sequences were used (for both comparisons, *P* < 0.0001 by two-tailed paired *t*-test as well as Wilcoxon signed rank test).

**Fig 5:**
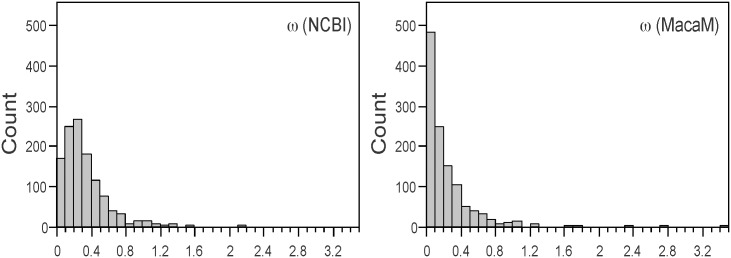
Distributions of ω estimated using the two rhesus gene annotations. Frequency distributions of ω values are shown for the 1,131 genes where the two rhesus genome annotations have different coding sequence annotations. ω' s were estimated using the rheMac2/NCBI (left panel) and MacaM (right panel) sequences compared against human orthologs.

**Fig 6:**
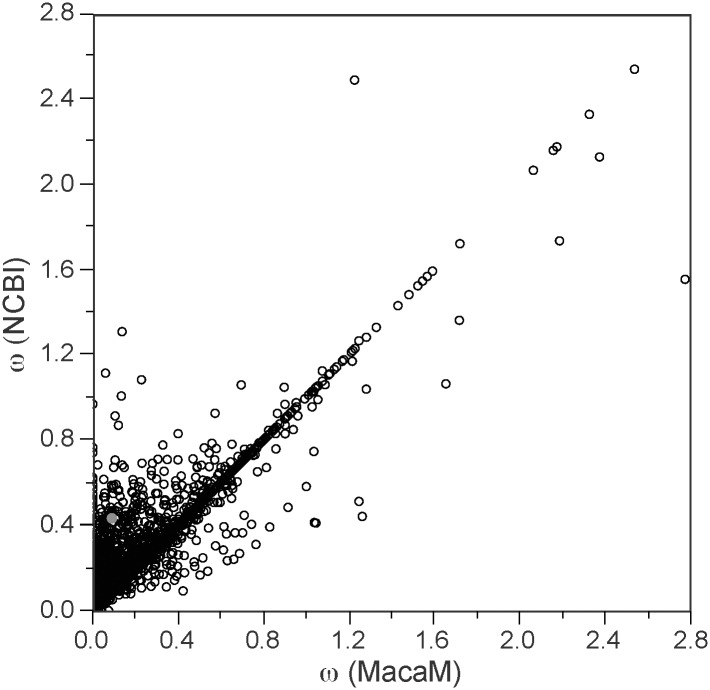
Comparison of ω estimated using the two rhesus gene annotations. All 3,606 ortholog pairs are included in the comparison. The red dot shows where the AGPAT4 is plotted (see Fig 2 for details).

Fig 5 shows the distribution of ω estimated from 1,131 human-rhesus ortholog pairs whose rhesus coding sequences were different between the rheMac2/NCBI (left panel) and MacaM (right panel) annotations. The mean ω’s were higher when the rheMac2/NCBI sequences were used (0.302±0.221) compared to when the MacaM sequences were used (0.206±0.262). These distributions were significantly different (P < 0.0001 by both two-tailed *t*-test as well as Mann-Whitney *U* test). The ω from the entire dataset was compared in Fig 6. The rheMac2/NCBI genome annotation indicated significantly higher values of ω (mean ω = 0.231±0.243) than MacaM (mean ω = 0.201±0.250) (P < 0.0001 by two-tailed paired *t*-test as well as Wilcoxon signed rank test).

Recently, a new assembly of the rhesus genome has been released by NCBI. This assembly (Mmul_8.0.1, release on 2015/11/30), is based on the MacaM assembly with some gaps filled with PacBio reads. We compared the NCBI annotation of this assembly (Release 102) with our MacaM annotation. Among the 3,606 genes we analyzed, we found 352 coding sequences to be different from our MacaM sequences. We calculated ω’s using these Mmul_8.0.1 sequences compared against the human orthologs. As shown in S1 Fig, for the same set of 352 sequences, ω’s obtained based on the rheMac2/NCBI sequences were significantly different (higher) from those obtained using the MacaM sequences, while when the Mmul_8.0.1 sequences were used, ω values obtained were very similar to those based on the MacaM sequences.

## Discussion

In interpreting the results we obtained, we made the assumption that when two rhesus macaque sequences from different annotations differed from each other, the one most similar to the human sequence was most likely to be correct. We think it unlikely that an incorrect rhesus sequence would, by chance, be more similar to the human sequence than a correct sequence. Thus, we conclude that long stretches of rheMac2 sequence that do not align with human or MacaM sequence are likely spurious and that elevated dN, dS, and ω values obtained with the rheMac2 genome but not with MacaM are likely due to incorrect rheMac2 sequences. An ω close to or higher than 1 indicates the gene is under positive selection. Therefore, our study demonstrates that use of the improved MacaM genome annotation greatly decreases the number of genes erroneously predicted as under positive selection if the rhemac2 genome was used.

We have previously provided examples of how sequencing errors and misassemblies in the rheMac2 assembly resulted in incorrect gene models [2, 3]. Further, we demonstrated how such errors were corrected in MacaM [12]. Here, we showed how one such error in rheMac2, in the AGPAT4 gene, can affect interpretations of the evolution of this gene. If the rheMac2 version of AGPAT4 were taken at face value, one would incorrectly conclude this gene to be either under relaxed negative selection or potentially under positive selection. Given that the MacaM gene model is correct, AGPAT4 is actually highly conserved (ω is close to 0). The summary statistics we provided indicated this problem is pervasive.

In the paper describing the rheMac2 assembly [9], a set of orthologs among humans, rhesus, and chimpanzees were identified for evolutionary analyses. However, many genes were rejected for this set due to problems in the nonhuman primate assemblies or annotations [9]. Thus, the problems associated with using the draft rhesus genome for evolutionary comparisons were recognized by the original submitters of this genome. After filtering out gene models they considered incorrect, they determined that: “The average human gene differs from its ortholog in the macaque by 12 nonsynonymous and 22 synonymous substitutions…” We observed mean (and median) values of 14.14 (8.53) nonsynonymous and 30.64 (23.75) synonymous substitutions with MacaM but 63.90 (12.04) nonsynonymous and 86.35 (26.39) synonymous substitutions with rheMac2. It is not clear whether “the average human gene” refers to a mean or median calculation. If this value refers to a mean, the synonymous and nonsynonymous values obtained in the paper describing rheMac2 [9] were close to our calculations using MacaM (but not rheMac2). If a median was calculated, the numbers obtained were consistent with both our calculations. As noted in S1 Table, internal stop codons were identified in 85 of the 3,606 genes we examined for the rheMac2/NCBI genome. These internal stop codons were treated as irregular "non-stop" codons and the genes were treated as protein-coding. As mentioned before, many rhesus genes appear to have significantly different lengths compared to human orthologs when the rheMac2 genome was used. It is likely that these genes were filtered out from the rheMac2 genome analysis. We have demonstrated that more accurate evolutionary comparisons can be performed on many more genes with MacaM than rheMac2, without any ad hoc filtering. It is also important to note that the rheMac2 genome is similar in quality to many published draft genomes, raising concerns about the use of these genome data for further, especially for genome-wide, analyses.

In this work, we used the rheMac2 assembly with the NCBI annotation as our exemplar of the draft rhesus genome. However, as we noted before, the Ensembl annotation of the rheMac2 assembly also results in errors similar to those produced by the NCBI pipeline. We have also documented the scope of errors created by Ensembl annotations in the draft genomes of two additional nonhuman primates: marmosets [22] and chimpanzees [23]. In a previous work [12], we also analyzed the quality of the CR_1/rheMac3 genome produced by the BGI [21]. Specifically, we noted that the CR_1 assembly was more fragmented than MacaM or rheMac2 (Table 4 in [12]). We demonstrated that CR_1 had annotations that were also defective (Fig 3 in [12]). The newest rhesus assembly version recently released by NCBI, Mmul_8.0.1, improved the rhesus genome assembly by incorporating the MacaM assembly. Since Mmul_8.0.1 is largely based on MacaM_v7, one would expect that it would be similarly superior to rheMac2. We found this to be true with respect to the estimated ω’s.

Our evolutionary analysis in this study is based only on pairwise alignments. Misaligning wrong exon regions or non-orthologous sequences will have further compounding effects on evolutionary analysis when multiple sequences are aligned. It should also be noted that evolutionary rate based analysis (dN, dS, and ω) requires sequences to be aligned. As shown in the example alignment in Figs 1 and 2, when long exons are incorrectly inserted or omitted, such regions are often entirely or partially aligned against gaps and largely ignored from calculation of evolutionary rates. Thus, our analyses provide only a glimpse of the adverse effects of using incorrect rhesus gene annotations in analyses of primate evolution.

Although we were focused on the rhesus macaque in the current work, it is important to note that the issues identified with this draft genome apply to other nonhuman primate draft genomes as well. To gain maximum understanding of selection in humans, it is necessary to compare genes among multiple species of nonhuman primates. Given that approximately 50% of gene annotations in rhesus macaques are missing, incomplete, or wrong [2], the problems identified here are compounded when more species are examined as genes that might be correctly annotated in two species might not be correct in a third, fourth, or fifth species. Indeed, in the original paper describing the draft rhesus macaque genome, the authors report that only 10,376 genes could be compared among humans, chimpanzees, and rhesus macaques due to errors in assembly or annotation in the chimpanzee and rhesus macaque genomes [9]. They did in fact find that there were many genes that might have been correct in chimpanzees that were not correct in rhesus macaques and *vice versa*. This finding provides motivation to improve the quality of multiple nonhuman primates to MacaM level.

One of the most important scientific questions ever posed is: how did humans evolve their unique characteristics, especially as regards cognition? The expectation that this question would be answered as a result of sequencing nonhuman primate genomes has only partially been realized. We, and others [1, 8], have demonstrated that this goal has been impeded by inadequate nonhuman primate genomes. Errors in sequences, assemblies, and annotations have created a plethora of false positives and left many other genes unexamined. The MacaM rhesus genome, although by no means perfect, greatly reduces the amount of noise created by error and increases the number of genes that can be studied. Allocation of resources to upgrade draft genomes, even after their publication, to the much higher quality of MacaM, especially for primate species, is justified. If the approach taken by our group to improve rhesus macaque genome quality is applied to other nonhuman primates, we anticipate that important new discoveries related to human evolution will be made as the signals of selection begin to rise above the noise of error.

## Supporting Information

S1 Fig Comparisons of ω estimated using the three rhesus gene annotations (rheMac2, Mmul_8.0.1, and MacaM). (PDF)

S1 Table List of all 3,606 genes used in this study. (XLSX)
