# Supplementary material for: Advantages of an Improved Rhesus Macaque Genome for Evolutionary Analyses
Source: PLoS One. 2016 Dec 2;11(12):e0167376. doi: 10.1371/journal.pone.0167376 (PMC5135103; doi:10.1371/journal.pone.0167376)
Supplement: S1 Fig — (PDF) [file pone.0167376.s001.pdf]

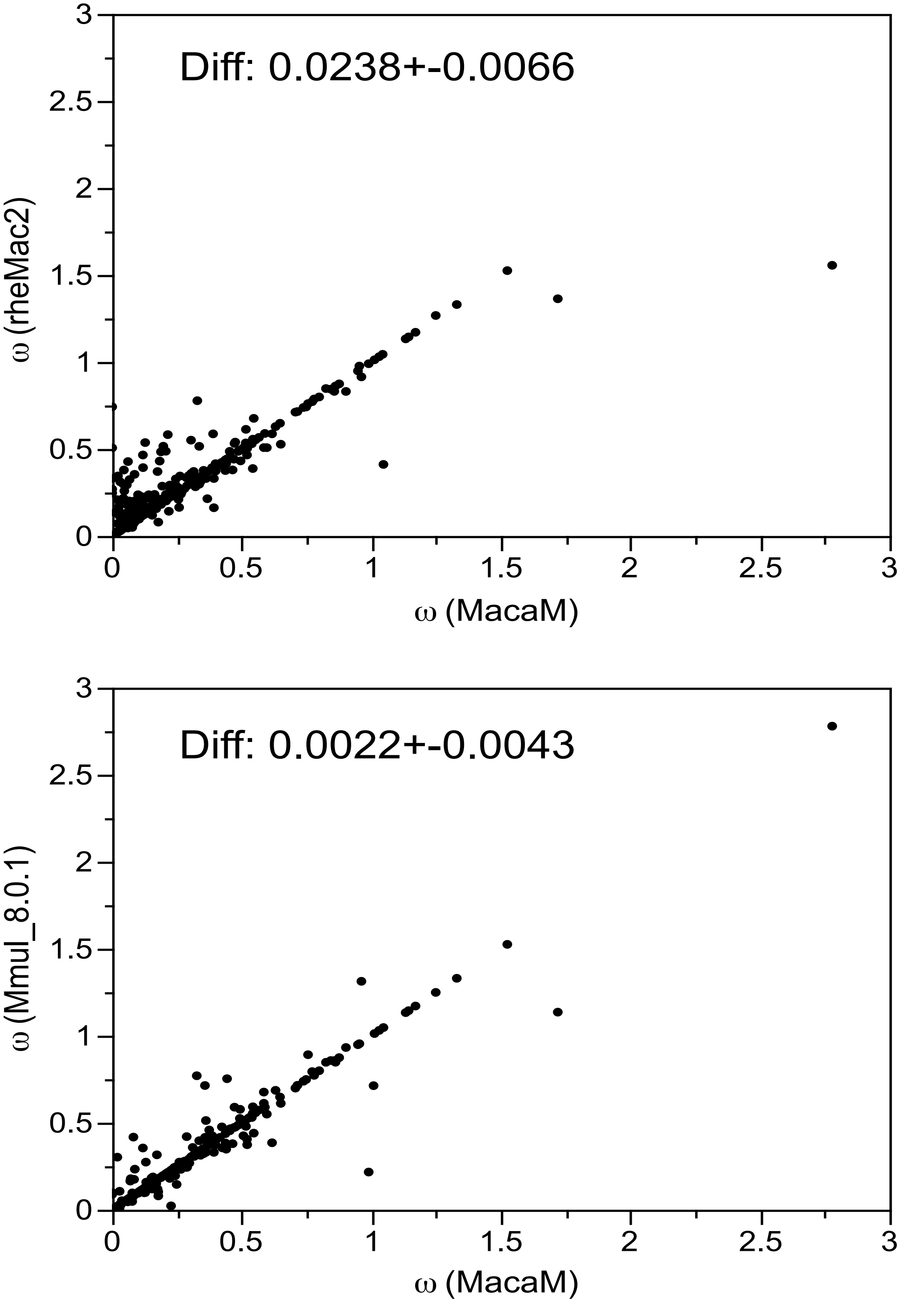

Fig S1. Comparisons of ω estimated using the three rhesus gene annotations (rheMac2, Mmul_8.0.1, and MacaM). The coding sequences of 352 genes where MacaM and Mmul_8.0.1 sequences were different were compared against human orthologs. The mean and standard deviation of differences in ω between rheMac2 and MacaM (top) and between Mmul_8.0.1 and MacaM (bottom) are shown in each panel ("Diff").
